# Combined therapy of human amnion-derived mesenchymal stem cells and scalp acupuncture alleviates brain damage in a rat model of cerebral palsy

**DOI:** 10.1016/j.ibneur.2024.12.015

**Published:** 2025-01-02

**Authors:** Yu Zhou, Xu-Huan Li, Lu-Na He, Li-Na Wang, Jing Zang, Dong-Ming Wang, Jing Gao, Xue-feng Yu

**Affiliations:** a Department of Pediatric Rehabilitation, Huai’an Maternal and Child Health Care Hospital Affiliated to Yangzhou University Huai’an, Jiangsu 223021, China; b Department of Orthopedics, Affiliated Rehabilitation Hospital, Jiangxi Medical College, Nanchang University, Nanchang, Jiangxi 330003, China; c Department of Orthopedics, The Fourth Affiliated Hospital of Nanchang University, Nanchang, Jiangxi 330003, China

**Keywords:** Cerebral palsy, Hypoxic-ischemic encephalopathy, Mesenchymal stem cells, Scalp acupuncture, Apoptosis

## Abstract

**Background:**

Cerebral palsy (CP) is a prevalent cause of physical disability in children, often resulting from hypoxic-ischemic encephalopathy, with current therapies often failing to address the underlying pathophysiology. This study aimed to investigate the potential synergistic effects of human amnion-derived mesenchymal stem cells (hAMSCs) combined with scalp acupuncture in a rat model of CP.

**Methods:**

Twenty male Sprague-Dawley rats were randomly divided into four groups: Sham, CP, hAMSCs, and hAMSCs+scalp acupuncture (hAMSCs+AP). The CP model was induced via left common carotid artery ligation. hAMSCs were administered through tail vein injection, followed by scalp acupuncture at Baihui (GV20) and Qubin (GB7) points. Neurobehavioral function was assessed using the Bederson score, and brain tissues were analyzed using hematoxylin and eosin (H&E) staining, TUNEL staining, and RT-qPCR for apoptosis-related genes.

**Results:**

The CP group exhibited significant neurobehavioral deficits and increased apoptosis. Both hAMSCs and hAMSCs+AP treatments improved neurobehavioral function and reduced apoptosis. The combination therapy further decreased apoptosis levels, normalized mRNA expression of Bax, Caspase 9, and Caspase 3, and alleviated histological damage.

**Conclusions:**

The combination of hAMSCs and scalp acupuncture provides a promising treatment for CP, potentially alleviating brain damage through apoptosis regulation. Further studies are required to elucidate the detailed mechanisms and assess clinical feasibility and safety.

## 1 Introduction

Cerebral palsy (CP) occurs in two to three out of every 1000 live births and is a common cause of physical disability in children(Vitrikas et al., 2020). Although the exact etiology of CP is complex, hypoxic-ischemic encephalopathy is a well-established cause. Hypoxic-ischemic brain injury is often used to construct CP animal models(Wu et al., 2022). Current physical therapy in clinical practice does not address the underlying causes of CP, particularly in severe cases. Therefore, novel treatment methods are urgently needed.

Studies have indicated that stem cell therapy can help repair damaged neurons and provide immunomodulation, pro-angiogenesis, and neuroprotective functions for hypoxic-ischemic brain damage(Jia et al., 2022). Human amnion-derived mesenchymal stem cells (hAMSCs) are a type of mesenchymal stem cell derived from the human placenta(Li et al., 2019). Numerous studies have shown that MSCs can promote angiogenesis in ischemic brain regions through anti-apoptosis mechanisms and the regulation of immune responses(Ulpiano et al., 2021; Van Nguyen et al., 2021). However, the therapeutic efficiency of stem cell treatments is limited by the complex microenvironment following ischemia-reperfusion injury, which hampers intracerebral migration of the stem cells.

Acupuncture has been utilized in clinical practice in China for more than 3000 years(Zhuang et al., 2013). Jingluo is a system of internal main and collateral channels, regarded as a network of energy passages. Numerous specific points in the Jingluo network, known as acupoints, can be stimulated through needling to excite energy within Jingluo, thereby improving the internal environment and mitigating pathogenic damage in stroke(Zheng et al., 2016). Acupuncture has demonstrated neuroprotective effects, maintained blood-brain barrier integrity, and inhibited apoptosis in cerebral ischemia(Chang et al., 2018; Zhu et al., 2017).

Our previous study showed that hAMSC injection could partially improve a CP rat model(Zhou et al., 2024). Scalp acupuncture has also been shown to provide neuroprotection and regulate immune inflammation in cerebral ischemia, including CP, in some clinical studies(Wan et al., 2016; Xu and Tong, 2023). However, no studies have explored the combination of stem cell therapy and acupuncture for CP treatment. Therefore, we aimed to investigate whether the combination of hAMSCs and scalp acupuncture treatment has a synergistic effect on a hypoxia/ischemia-induced CP rat model.

## 2 Methods

### 2.1 Animals

The study protocol was approved by the Ethics Committee of Huai’an Children and Maternal and Child Health Care Center (No. 202168). Twenty male Sprague-Dawley rats, weighing 220–280 g, were used in this study. The rats were housed at room temperature (25 ± 2°C) with 55 ± 5 % humidity under a 12-hour light–dark cycle, with free access to water and food. All experimental protocols and procedures were performed in accordance with the Guidance Suggestions for the Care and Use of Laboratory Animals, issued by the Ministry of Science and Technology of China.

After acclimatizing to the housing facility for 7 days, the rats were randomly divided into four groups (5 rats in each group): Sham group, CP group, hAMSCs group, and hAMSCs + scalp acupuncture group (hAMSCs+AP). The CP rat model was induced as in previous studies by left common carotid artery ligation(Yu et al., 2014; Zhou et al., 2024). After intraperitoneal anesthesia with pentobarbital, an incision was made in the middle of the neck, and the left common carotid artery was exposed. A 5–0 silk thread was ligated between the upper and lower ends of the left common carotid artery and then cut off between the ligatures.

The hAMSCs were obtained from Procell company (H246, China) and identified by immunofluorescence. hAMSCs were cultured in cell culture dishes (Corning, NY, USA) at a density of 5 × 10^4 cells/cm^2 at 37 °C in a 5 % CO2 atmosphere. Twenty-four hours after establishing the CP model, 200 μL of hAMSCs (5 ×10^5 cells/100 µL) were injected through the tail vein in the hAMSCs group.

**Fig. 1:**
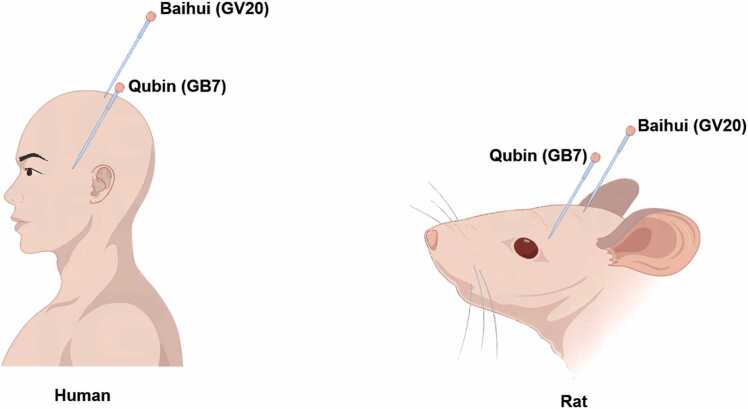
The locations of the Baihui and Qubin acupoints in humans and rats. Baihui is situated at the midpoint of the line between the ear tips, while Qubin is located at the 2/3 point along the line from the orbital margin to the external ear hole.

Rats in the Sham group underwent the same procedure as the CP group, except for the carotid artery ligation, and received an equal volume of normal saline. In the hAMSCs+AP group, rats were administered scalp acupuncture after the stem cell injection. The acupoints in the rats were positioned along the temporal anterior and parietal anterior oblique, corresponding to human acupuncture points, named Baihui (GV20) and Qubin (GB7) (as shown in Fig. 1). The acupuncture needles inserted 2 mm into the acupoint, and connected to an electro-acupuncture therapeutic apparatus (BT701–1B, HuaYi, China), delivering a bidirectional pulse wave (3.5 mA, 2 Hz, 0.5 ms), once per day, for 30 minutes each time, over a period of 14 days. All rats were anesthetized with pentobarbital sodium and euthanized by carbon dioxide suffocation 14 days after the corresponding treatment.

### 2.2 Identification of hAMSCs by flow cytometry

To analyze the phenotype of cultured hAMSCs, flow cytometry was conducted as previously outlined (Li et al., 2019). The cells were collected at a concentration of 1 × 10^6 cells/mL in staining buffer and placed in fluorescence-activated cell sorting (FACS) tubes (BD Biosciences, Franklin Lakes, NJ). The hAMSCs were then incubated with a panel of antibodies: PerCP-conjugated anti-CD34, PE-conjugated anti-CD73, PE-Cy7-conjugated anti-CD44, and FITC-conjugated anti-CD90, along with their respective isotype controls (all from BD Biosciences). The staining process was carried out in the dark at 4°C for 30 minutes. Following two washes, the cells were resuspended in 200 μL of PBS for analysis using a FACSCalibur flow cytometer (BD Biosciences). Data were subsequently analyzed using FLOWJO™ software (TreeStar, Inc., Ashland, OR, USA).

### 2.3 Neurobehavioral function assessment

The Bederson neurological function score was evaluated by an independent researcher who was blinded to the experimental groups (Bederson et al., 1986). All rats were assessed before being sacrificed according to the following standard(Li et al., 2010): 0 score: No neurological functional impairment. 1 score: Any part of the forepaw flexed (positive for the tail suspension test) without other abnormal signs. 2 scores: Decreased resistance to lateral pushing (positive for the lateral pushing experiment), accompanied by forepaw flexion without a circling tendency. 3 scores: The same behaviors as those for 2 scores, in addition to spontaneous rotation (circling around paralyzed limbs during free activity). A higher score represents worse neurobehavioral dysfunction.

### 2.4 Hematoxylin and eosin (H&E) and terminal deoxynucleotidyl transferase dUTP nick end labeling (TUNEL) staining

The brain hippocampal tissues from each group were fixed in 4 % paraformaldehyde, dehydrated, embedded in paraffin, and then cut into 4-μm slices. All slices were deparaffinized, hydrated, washed, and stained according to a standard protocol as previously described(Wei et al., 2023). Morphological changes were observed under a microscope (BX43, OLYMPUS). TUNEL staining was conducted using a TUNEL apoptosis assay kit (C1090, Beyotime). TUNEL-positive cells were stained with red fluorescence, and nuclei were counterstained with blue using DAPI. Ten randomly selected high-power fields were analyzed by a researcher blinded to the experimental groups.

### 2.5 Extraction of total RNA and real‑time quantitative PCR

Samples (50 mg) from each group were mixed with 1 mL TRIzon Reagent after grinding, according to the instructions of the RNA extraction kit (W0581M, CWBIO). One microgram of RNA was used for reverse transcription to synthesize cDNA, following the manufacturer's protocol for the reverse transcription kit (R223–01, Vazyme). The primers used are listed below. NADPH was used as the internal reference. The relative mRNA expression was evaluated using the 2 −ΔΔCT method. Each assay was performed in triplicate.

### 2.6 Primer sequences for the target genes

**Table:** 

Gene		Primer sequence (5′–3′)
Bax	Forward	GCGATGAACTGGACAACAAC
	Reverse	GCAAAGTAGAAAAGGGCAACC
Bcl−2	Forward	GCGTCAACAGGGAGATGTCA
	Reverse	TTCCACAAAGGCATCCCAGC
Caspase 9	Forward	GGGAAGATCGAGAGACATGCAGATA
	Reverse	AGCCGTGACCATTTTCTTAGCAG
Caspase 3	Forward	CTAAGGAAGATCACAGCAAAAGG
	Reverse	TAGAGTAAGCATACAGGAAGTCGG
GAPDH	Forward	GACAACTTTGGCATCGTGGA
	Reverse	ATGCAGGGATGATGTTCTGG

### 2.7 Immunofluorescence (IF) identification

The hAMSCs were grown on glass coverslips and rinsed with PBS three times. The cells were fixed in 4 % paraformaldehyde for 15 minutes. Then, 5 % bovine serum albumin was used as a blocking solution at 37°C for 30 minutes. The cells were incubated with antibodies against CD44 (1:200, DF6392, Affinity), CD90 (1:400, A12623, ABclonal) and CD45 (1:200, A23549, ABclonal) overnight at 4°C. Subsequently, the slices were incubated with Cy3 (1:200, AS007, ABclonal) at 37°C for 30 minutes. The nuclei were then stained with DAPI for 3 minutes and observed using a fluorescence microscope (Ckx53, Olympus).

### 2.8 Statistical analysis

All data were presented as the means ± SEMs. Statistical analysis was performed using GraphPad Prism 7.0 software. Group comparisons were performed using a two-tailed Student’s *t*-test (for two groups) or one-way analysis of variance (ANOVA) followed by a post hoc test (Tukey's Honestly Significant Difference (HSD) test). A P value < 0.05 was considered to indicate statistical significance.

## 3 Results

### 3.1 Immunofluorescence Identification of hAMSCs and Neurobehavioral Assessments

**Fig. 2:**
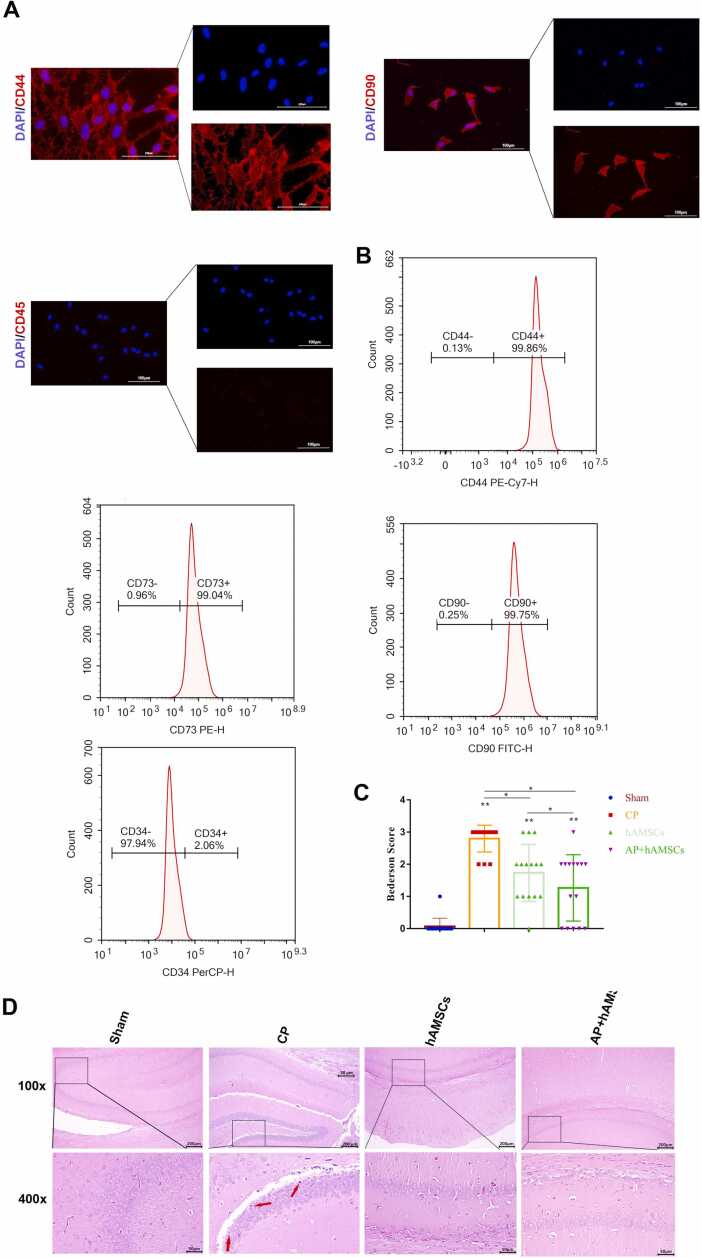
Effect of combination therapy of hAMSCs and acupuncture on neurobehavioral assessment and pathological changes. (A) Representative fluorescence microscopy images showing the expression of CD44, CD90, and CD45 in mesenchymal stem cells (red). Nuclei were counterstained with DAPI (blue). Scale bar = 100 μm. (B) Flow cytometry analysis of mesenchymal stem cells showing positive markers (CD44, CD73, and CD90) and a negative marker (CD34). (C) Assessment of neurobehavioral function based on the Benderson score. (D) Representative hematoxylin and eosin (H&E)-stained hippocampal sections from the different groups. Dead cells are indicated by red arrows. Scale bar = 50 μm. All data are presented as the mean ± SEM. Statistical significance is indicated as follows: * p < 0.05; * * p < 0.001.

hAMSCs, a type of mesenchymal stem cell, were identified by immunofluorescence staining and flow cytometry. The cells exhibited positive expression for CD44 and CD90 (red) and negative expression for CD45. Nuclei were counterstained with DAPI, appearing blue (Fig. 2A). Flow cytometry further confirmed that hAMSCs expressed mesenchymal stem cell markers CD44, CD73, and CD90, while lacking expression of the hematopoietic stem cell marker CD34 (Fig. 2B).

Bederson scores indicated significant deficits in the CP group after left common carotid artery ligation. After the injection of hAMSCs in CP rats, Bederson scores decreased, indicating improved neurobehavioral function. Rats in the AP+hAMSCs group showed marked improvement in neurobehavioral function compared to both the CP and hAMSCs groups (Fig. 2C).

### 3.2 Morphological Changes After hAMSCs and Scalp Acupuncture Treatment

H&E staining revealed significant morphological differences among the groups. In the Sham group, hippocampal tissues exhibited intact neuronal architecture without noticeable damage. In contrast, the CP group demonstrated severe pathological changes, including extensive neuronal death and vacuolization (indicated by red arrows), the hippocampal regions showed disrupted architecture and reduced neuronal density, indicative of significant pathological damage, indicative of substantial brain injury. Treatment with hAMSCs partially alleviated these damages, as evidenced by reduced neuronal loss and improved structural integrity. Notably, the combined therapy of hAMSCs and scalp acupuncture (AP+hAMSCs) resulted in a pronounced restoration of hippocampal morphology, with minimal neuronal damage and vacuolization, closely resembling the Sham group (Fig. 2D). These findings highlight the potential therapeutic synergy of hAMSCs and acupuncture in alleviating cerebral palsy-induced brain damage.

### 3.3 Apoptosis Level Tendency in Different Groups

**Fig. 3:**
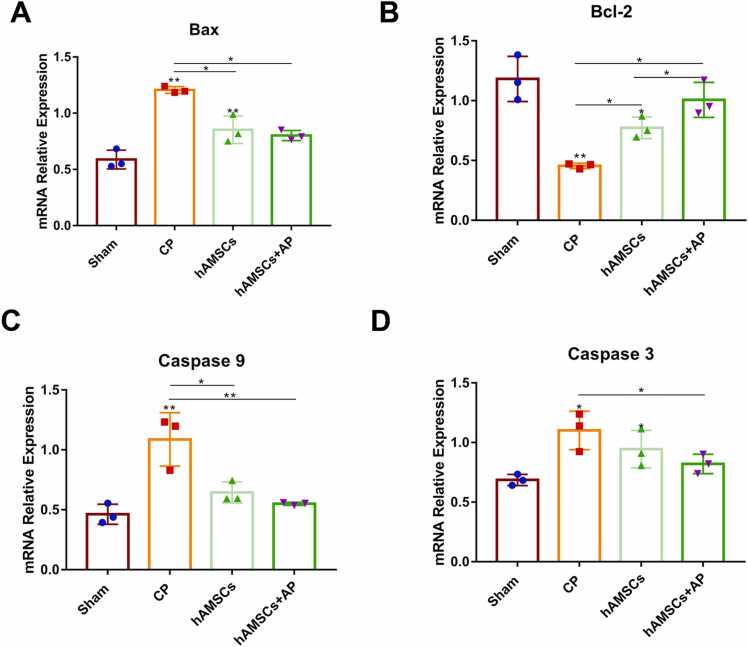
The mRNA expression of apoptotic genes in each group. Relative mRNA expression levels of Bax, Bcl-2, Caspase 9, and Caspase 3 in the Sham, CP, hAMSCs, and hAMSCs + AP groups. All data are presented as the mean ± SEM. Statistical significance is indicated as follows: * p < 0.05; * * p < 0.001.

The changes in apoptosis levels were assessed using RT-qPCR and TUNEL staining. Compared to the Sham group, the CP group exhibited significantly increased mRNA levels of Bax, Caspase 9, and Caspase 3 (p < 0.05), whereas Bcl-2 levels were significantly decreased (p < 0.05). In the hAMSCs group, the mRNA expression levels of Bax, Bcl-2, and Caspase 3 showed similar trends as those observed in the CP group when compared to the Sham group (p < 0.05). However, Caspase 9 levels in the hAMSCs group were significantly reduced compared to the Sham group (p < 0.05). In the hAMSCs+AP group, the mRNA levels of Bax, Caspase 9, and Caspase 3 were decreased, and Bcl-2 levels did not differ significantly from those in the CP group (p > 0.05) (Fig. 3).

**Fig. 4:**
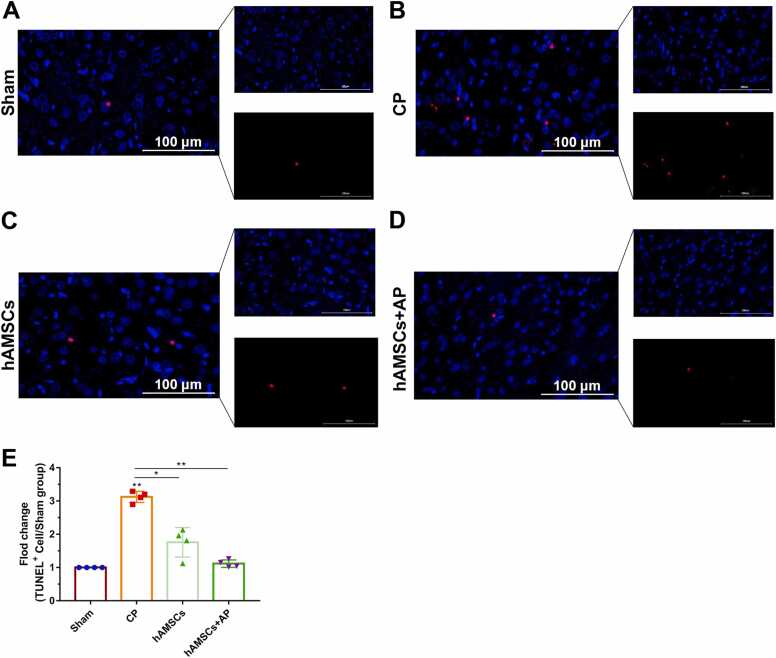
TUNEL staining of apoptotic cells in the brains of mice with cerebral palsy. (A-D) Representative TUNEL staining of apoptotic cells from randomly selected mice in each group. (E) The fold change of TUNEL-positive cells in different groups relative to the Sham group. All data are presented as the mean ± SEM. Statistical significance is indicated as follows: * p < 0.05; * * p < 0.001.

The CP group demonstrated a significant increase in the number of TUNEL-positive cells compared to the Sham group. There was no significant difference in the percentage of apoptotic cells between the hAMSCs and hAMSCs+AP groups when compared to the Sham group (p > 0.05). Although the number of TUNEL-positive cells did not differ between the hAMSCs and hAMSCs+AP groups, apoptosis levels in the hAMSCs+AP group more closely resembled those of the Sham group (Fig. 4). The detailed information of ANOVA analysis was provided in Table S1.

## 4 Discussion

Cerebral palsy caused by hypoxic-ischemic encephalopathy presents a challenging scenario in clinical practice. In the present study, both hAMSCs and hAMSCs+AP treatments were found to alleviate brain damage. Scalp acupuncture combined with hAMSCs may provide a better therapeutic effect than hAMSCs treatment alone by mitigating apoptosis in CP rats.

Endogenous mesenchymal stem cells (MSCs) mobilize into the peripheral circulation following brain ischemia. Numerous studies on MSC injection treatments have demonstrated strong immunomodulatory and anti-neuroinflammatory effects for hypoxic-ischemic encephalopathy(Li et al., 2016; Qin et al., 2019). hAMSCs treatment can facilitate the regeneration of endogenous nerve cells in brain injury tissue(Zheng et al., 2021). Our findings also indicate that hAMSCs injection promotes neurobehavioral improvement and recovery of pathological lesions. However, the low survival and differentiation rates of these cells limit their therapeutic value, making this a significant focus of current research.Acupuncture is an alternative and complementary method for ischemic encephalopathy recommended by the World Health Organization(Chavez et al., 2017). According to Chinese medicine classification, cerebral palsy is recognized as “five delays and five softness, hand and foot cramps.” The Baihui acupoint, located at the governor vessel, can enhance memory via long-term potentiation (LTP) recovery in the hippocampus(Lin and Hsieh, 2010). Qubin is another crucial acupoint for addressing mental retardation. Needle insertion at “Baihui” and “Qubin” increases levels of brain-derived neurotrophic factor (BDNF) and improves functional and motor recovery after ischemic stroke(Kim et al., 2012).

The combination therapy of electroacupuncture and mesenchymal stem cells has demonstrated synergistic effects in promoting neurological function recovery and angiogenesis in rats following cerebral ischemia/reperfusion(Yu et al., 2014). In the present study, scalp acupuncture at the “Baihui” and “Qubin” points following hAMSCs injection alleviated brain damage in CP rats, as evidenced by neuroethological assessments and histological observations. Other studies also indicate that stimulation of the “Baihui” point can reduce the inflammatory response in the ischemic brain(Wang et al., 2023). Treatment involving “Baihui” and “Qubin” has been shown to decrease neurobehavioral deficits and inhibit apoptotic cell death(Guan et al., 2021).

It has been found that acupuncture and stem cell transplantation can exert complementary effects. In the present study, the excessive apoptosis levels in the brain were normalized in the hAMSCs+AP group compared to the hAMSCs group, as detected by RT-qPCR and TUNEL staining. Acupuncture has been shown to enhance TrkB-MSCs' therapeutic effects and promote the differentiation of TrkB-MSCs into mature neurons in an ischemic stroke model(Ahn et al., 2019). Consistent with our findings, previous research also demonstrates that combination therapy can inhibit cellular apoptosis and promote VEGF expression in ischemic diseases(Yang et al., 2013).

We hypothesize that acupuncture modifies the local microenvironment in the damaged brain, such as increasing cerebral blood flow and restoring the blood-brain barrier(Jin et al., 2023). hAMSCs exert neurotrophic and immunomodulatory effects. Both treatments perform compensatory and complementary functions, enhancing the therapeutic effects of hAMSCs for cerebral palsy.

However, there were some limitations in this study. There is no standard protocol for electro-acupuncture in cerebral palsy or ischemic stroke animal models. The parameters for electro-acupuncture range from 1 mA to 3.5 mA, and the course of treatment varies from 7 days to 21 days(Liang et al., 2017; Luo et al., 2017; Tu et al., 2018; Zhang et al., 2020). Similarly, the dosage of MSCs treatment for brain diseases remains inconsistent. The distribution of stem cells following intravenous injection was not assessed in this study. However, previous research has demonstrated that MSCs possess the ability to home to damaged tissues in models of traumatic neural injuries and cerebral ischemia (Tam et al., 2023, Yang et al., 2024). In future studies, we plan to investigate the distribution of stem cells in a cerebral palsy animal model to further elucidate this mechanism. Additionally, while the present study detected the function of apoptosis, only some Bcl2-family genes were detected, it did not elucidate the detailed mechanisms by which acupuncture combined with hAMSCs therapy benefits cerebral palsy. Furthermore, there is a need to develop targeted migration methods for brain diseases, as systemically administered cells may cause adverse effects in other tissues.

In conclusion, the present study primarily demonstrates that the combination therapy of hAMSCs and acupuncture is a potential treatment for the CP rat model, potentially alleviating brain damage by regulating excessive apoptosis levels. However, further studies are necessary to investigate the detailed mechanisms and clinical investigations are required to explore the feasibility and safety of this treatment approach.

## Ethics approval and consent to participate

This study protocol was approved by the Ethics Committee of Huai’an Children and Maternal and Child Health Care Center (No. 202168).

## Funding

This study was financially supported by The 10.13039/501100001809National Natural Science Foundation of China (Grant No. 82360879), the 10.13039/501100004608Natural Science Foundation of Jiangsu Province (Grant No. BK20241850, NO. BK20230292), and The Science and Technology Development Plan of Jiangsu Province Traditional Chinese Medicine Commission (Grant No. MS2021070).

## Consent for publication

Not applicable.

## CRediT authorship contribution statement

**Lu-Na He:** Software, Resources, Investigation. **Li-Na Wang:** Writing – original draft, Visualization, Resources, Investigation. **Yu Zhou:** Writing – review & editing, Validation, Funding acquisition, Data curation, Conceptualization. **Xu-Huan Li:** Methodology, Investigation, Formal analysis. **Dong-Ming Wang:** Resources, Methodology, Investigation. **Xue-feng Yu:** Writing – review & editing, Supervision, Project administration, Funding acquisition, Conceptualization. **Jing Gao:** Writing – review & editing, Funding acquisition, Conceptualization. **Jing Zang:** Software, Resources, Investigation.

## Declaration of Competing Interest

The authors declare that the research was conducted in the absence of any commercial or financial relationships that could be construed as potential conflicts of interest.

## Data Availability

The datasets used and/or analyzed in this study are available upon reasonable request from the corresponding author.
