# Supplementary material for: Combined therapy of human amnion-derived mesenchymal stem cells and scalp acupuncture alleviates brain damage in a rat model of cerebral palsy
Source: IBRO Neurosci Rep. 2025 Jan 2;18:263–9. doi: 10.1016/j.ibneur.2024.12.015 (PMC11810711; doi:10.1016/j.ibneur.2024.12.015)
Supplement: Supplementary file 1 — Supplementary material [file mmc1.docx]

**Table S1. Original analysis results of ANOVA**

| Terms | F (DFn, DFd) | P value |
| --- | --- | --- |
| Bederson behavior assessment | F (1.727, 24.18) = 34.01 | P<0.0001 |
| Bcl-2 | F (3, 8) = 18.08 | P=0.0006 |
| Bax | F (1.726, 3.452) = 31 | P=0.0065 |
| Cleaved-caspase3 | F (1.824, 3.648) = 8.735 | P=0.0414 |
| Caspase9 | F (3, 8) = 14.35 | P=0.0014 |
| TUNEL | F (3, 12) = 63.89 | P<0.0001 |
